# A novel recessive mutation in the gene ELOVL4 causes a neuro-ichthyotic disorder with variable expressivity

**DOI:** 10.1186/1471-2350-15-25

**Published:** 2014-02-26

**Authors:** Hina Mir, Syed Irfan Raza, Muhammad Touseef, Mazhar Mustafa Memon, Muhammad Nasim Khan, Sulman Jaffar, Wasim Ahmad

**Affiliations:** 1 Department of Biochemistry, Faculty of Biological Sciences, Quaid-i-Azam University, Islamabad, Pakistan; 2 Department of Zoology, University of Azad Jammu and Kashmir, Muzafarabad, Pakistan; 3 Shifa College of Medicine, Shifa International Hospital, Sector H-8/4, Islamabad, Pakistan; 4 Army Medical College, National University of Science & Technology NUST), Islamabad, Pakistan

**Keywords:** Ichthyosis, Phenotypic variability, *ELOVL4*, Non-sense mutation

## Abstract

**Background:**

A rare neuro-ichthyotic disorder characterized by ichthyosis, spastic quadriplegia and intellectual disability and caused by recessive mutations in ELOVL4, encoding elongase-4 protein has recently been described. The objective of the study was to search for sequence variants in the gene *ELOVL4* in three affected individuals of a consanguineous Pakistani family exhibiting features of neuro-ichthyotic disorder.

**Methods:**

Linkage in the family was searched by genotyping microsatellite markers linked to the gene *ELOVL4*, mapped at chromosome 6p14.1. Exons and splice junction sites of the gene *ELOVL4* were polymerase chain reaction amplified and sequenced in an automated DNA sequencer.

**Results:**

DNA sequence analysis revealed a novel homozygous nonsense mutation (c.78C > G; p.Tyr26*).

**Conclusions:**

Our report further confirms the recently described ELOVL4-related neuro-ichthyosis and shows that the neurological phenotype can be absent in some individuals.

## Background

Elongation of Very Long chain fatty acids-4 (ELOVL4) is a member of a large family of fatty acid elongases (ELO), involved in biosynthesis of very long-chain fatty acid (VLCFAs) with chain length ≥24 carbon atoms. Fatty acid elongases add two-carbon extensions to existing fatty acid substrates to generate longer chained fatty acids [1]. Seven members of the ELOVL family (ELOVL1-7) have been identified in humans that differ from each other in their substrate specificity, tissue expression and developmental patterns [2-4]. These can be divided into two major groups including ELOVL1, 3, 6 and 7, which are suggested to be elongases of saturated and monounsaturated VLCFA and ELOVL2 and 5, which are involved in the elongation of polyunsaturated fatty acids (PUFA) [2]. It has been identified that ELOVL4 is active both in the elongation of saturated VLCFA and in the biosynthesis of polyunsaturated VLC-PUFA [5].

It was first identified that heterozygous mutations in the gene *ELOVL4* are responsible for a dominant form of Stargardt-like macular dystrophy (STGD3) [6]. Patients with heterozygous *ELOVL4* mutations exhibit macular degeneration, which is probably related to a deficiency of VLCFA-containing lipids in the retina [7]. Recently, Aldahmesh et al. [8] have shown recessive mutations in the gene *ELOVL4* in two children causing congenital ichthyosis, along with a profound neurological phenotype and spastic quadriplegia.

In the present study we have investigated a consanguineous Pakistani family displaying an autosomal recessive neuroichthyotic disorder. Genotyping using microsatellite markers showed linkage of the family to the gene *ELOVL4,* mapped on chromosome 6q14. Subsequently, sequence analysis of the gene revealed a novel homozygous nonsense mutation (p.Tyr26*).

## Methods

### Subjects

**Figure 1:**
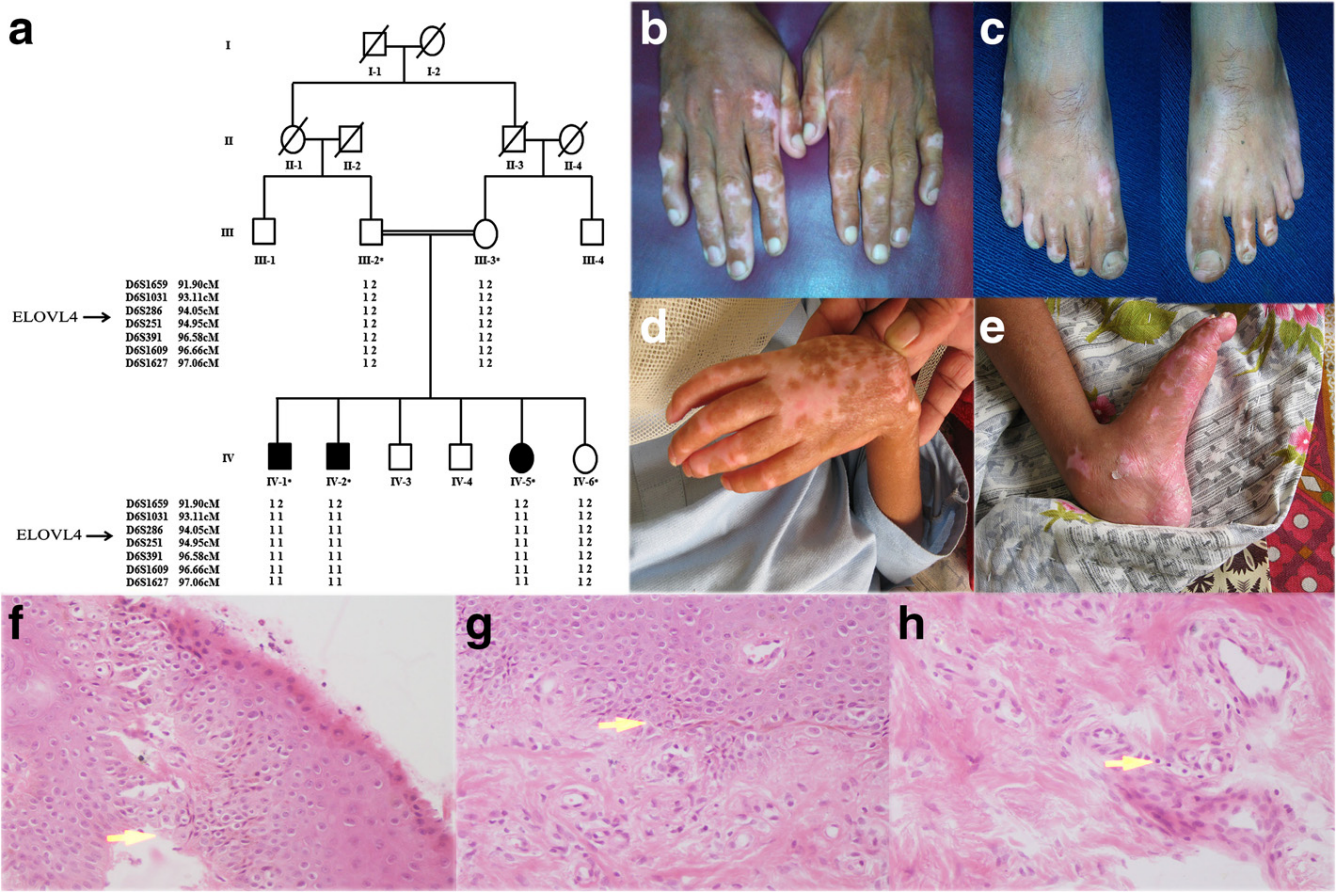
**Pedigree drawing of a consanguineous Pakistani family and clinical features observed in affected members. (a)** Pedigree drawing of a consanguineous Pakistani family with a neuro-ichthyotic disorder. Circles and squares represent females and males, respectively. Clear symbols represent unaffected individuals while filled symbols represent affected individuals. Symbols with crossed lines represent deceased individuals. Symbols with a star represent the samples that were available for the study. **(b-c)** clinical features of hereditary ichthyosis with dry, erythematous and hyperkeratotic skin on hands and feet of 18 year old affected individual IV-1; **(d-e)** and 14 year old affected individuals IV-2. **(f-h)** Histological examination of a skin biopsy of the affected individual IV-1 showed hyperkeratosis and acanthosis of the epidermis, with edema of the basal layer of epidermis and sparse lymphocytic cellular infiltrate scattered in the dermis mainly around the blood vessels. Informed written consent for the study and presentation of the photographs for publication was obtained from affected and unaffected individuals and their parents.

For the study, presented here, a four generation consanguineous family demonstrating autosomal recessive form of neuro-ichthyotic disorder was recruited from a remote region of Pakistan **(**Figure 1a). Approval of the study was obtained from the Institutional Review Board (IRB) of Quaid-i-Azam University, Islamabad Pakistan. Both affected and unaffected members of the family were informed about research methodology and objectives of this study. Pedigree drawing of the family was based upon detailed question/answer sessions from affected and elders of the family. Affected individuals IV-1, IV-2 and IV-5 were 24, 16 and 22 years old at the time of the study. The affected member IV-2 died recently at the age of 17 years.

Genomic DNA was extracted from peripheral blood samples collected from three affected and three unaffected members of the family by GenElute™ blood genomic DNA kit (Sigma-Aldrich, St. Louis, MO, USA). A skin biopsy was performed and the sample was fixed in 10% formalin and embedded in paraffin. Four-μm-thick sections were stained with hematoxylin-eosin.

### Genotyping

The family was tested for linkage by genotyping microsatellite markers linked to genes involved in neuro-ichthyotic syndromes. These include Aldehyde Dehyrogenase 3A2 gene (*ALDH3A2,* MIM 270200) on chromosome 17p11 (D17S261, D17S1857, D17S1794, D17S805, D17S842, D17S783, D17S1878), involved in Sjögren-Larsson Syndrome (SLS), and Elongase-4 gene (*ELOVL4,* MIM 605512) on chromosome 6p14.1 (D6S1659, D6S1031, D6S286, D6S251, D6S391, D6S1609, D6S1627). Order of markers was based on Rutgers combined linkage-physical map of the human genome [9].

### Sequencing

Standard sequence of the gene *ELOVL4* was obtained from Ensembl Genome Browser (http://asia.ensembl.org/index.html). Primers for PCR (polymease chain reaction) amplification of all six exons and splice junction sites of the gene were designed using Primer3 version 0.4.0 software [10]. DNA sequencing was performed with Big Dye Terminator v3.1 Cycle Sequencing Kit, together with an ABI Prism 310 Genetic Analyzer (Applera, Foster City, CA, USA). Sequence variants were identified via Bioedit sequence alignment tool (editor version 6.0.7, Ibis, Biosciences, CA, USA).

## Results

### Clinical features

All three affected individuals (IV-1, IV-2, IV-5) of the family, presented here, showed typical features of the hereditary ichthyosis. Skin in all the affected individuals was dry, erythematous and hyperkeratotic with scales present on lips, tip of the nose, ear pinna, legs, matacarpophalangeal joints and feet (Figure 1b-e). In individual IV-2, who died recently, back and neck were also affected. Intellectual disability and spastic quadriplegia were observed only in patient IV-2. He was born by normal vaginal delivery at home, but develops fever accompanied by frequent urination at 4 months of age. Profound developmental delay and occurrence of seizures were reported, a year after birth. Seizures were occurring very frequently mostly at twenty minutes time interval. Severe hypertonia developed in the upper and lower extremities in this individual. Bones and muscles appeared extremely weak. Speech and hearing were abnormal. He remained bed ridden until he died. Oral examination showed leukoplakia with white patches appeared on tongue. Significant dental erosion involving second incisor and canine were observed.

**Figure 2:**
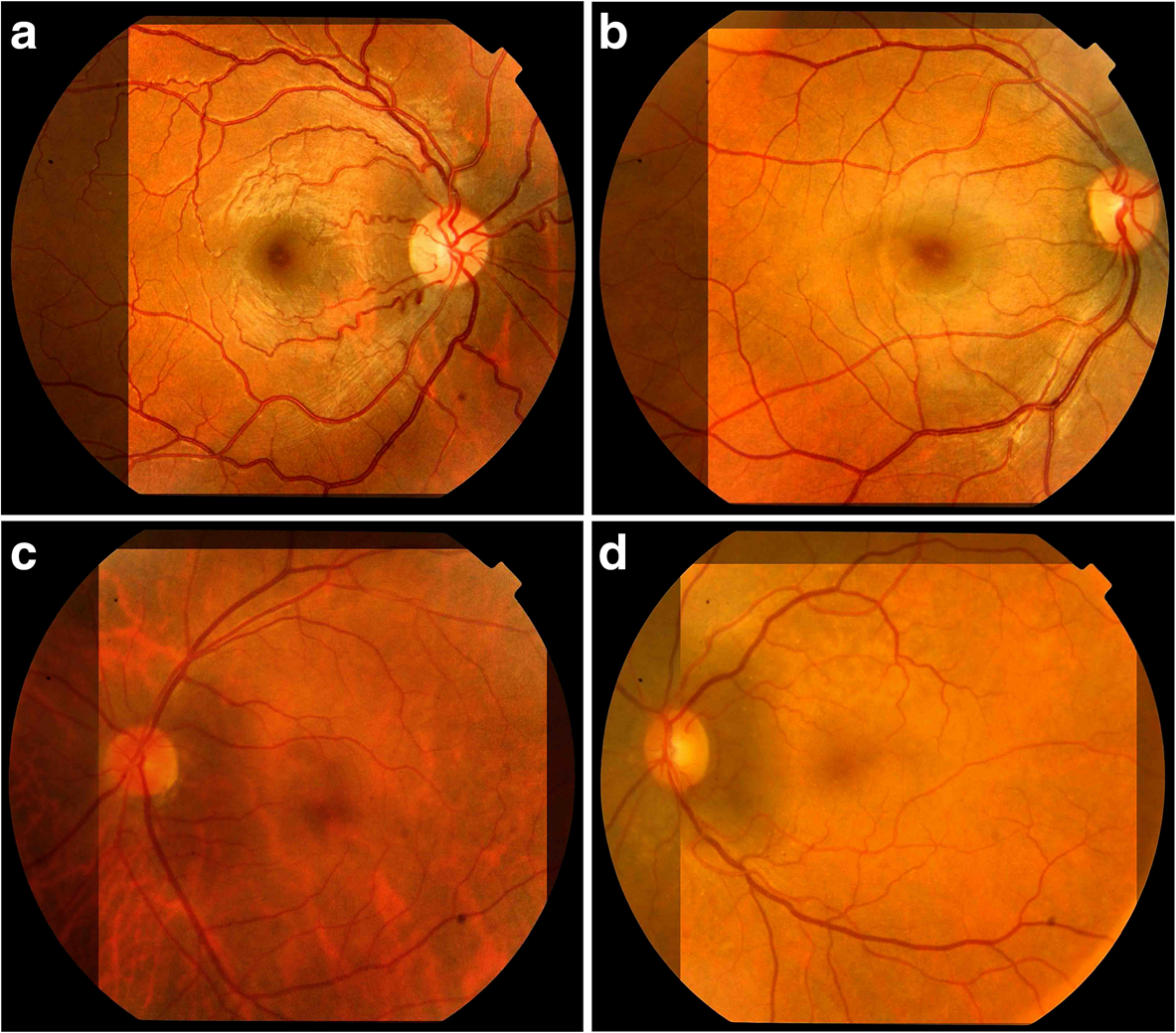
**Fundus examination of four individuals including two affected IV-1 (a) and IV-5 (b), father III-2 (c) and mother III-3 (d).** Note tortuous vessels in the macular area in the two affected individuals in panel **a** and **b**.

Fundus examination of four individuals (IV-1, IV-5, III-2, III-3) was carried out at Shifa Hospital Islamabad (Figure 2). Fundus examination of two affected individuals (IV-1, IV-5) showed no significant refractive error. However, tortuous vessels in the macular area with subtle macular changes were noted in these two individuals. In father (III-2) of the three affected children, the fundus examination revealed mild degree of myopia with subtle peripapillary changes. In mother (III-3) fundus examination was normal (III-3).

Skin biopsy of the affected individual IV-1 showed hyperkeratosis and acanthosis of the epidermis, edema of the basal layer of epidermis causing its disruption and leading to the formation of sub epidermal cleft formation (Figure 1). Sparse lymphocytic cellular infiltrate scattered in the dermis mainly around the blood vessels was noted.

In all the three affected individuals ichthyosis was present since birth. In cold weather, this condition turns to more severe form which develops itching and pain in the affected areas. Prominent ears with large pinna and yellow pigmented nails were observed in all the affected individuals.

Magnetic resonance imaging (MRI) and computed tomography (CT) scan images of the affected individuals were not available for the study. History of cardiac, diabetic and immunological problems was not reported in any of the affected individuals. Obligate heterozygous carriers in the family were normal and were clinically indistinguishable from genotypically normal individuals.

### Linkage and mutation analysis

**Figure 3:**
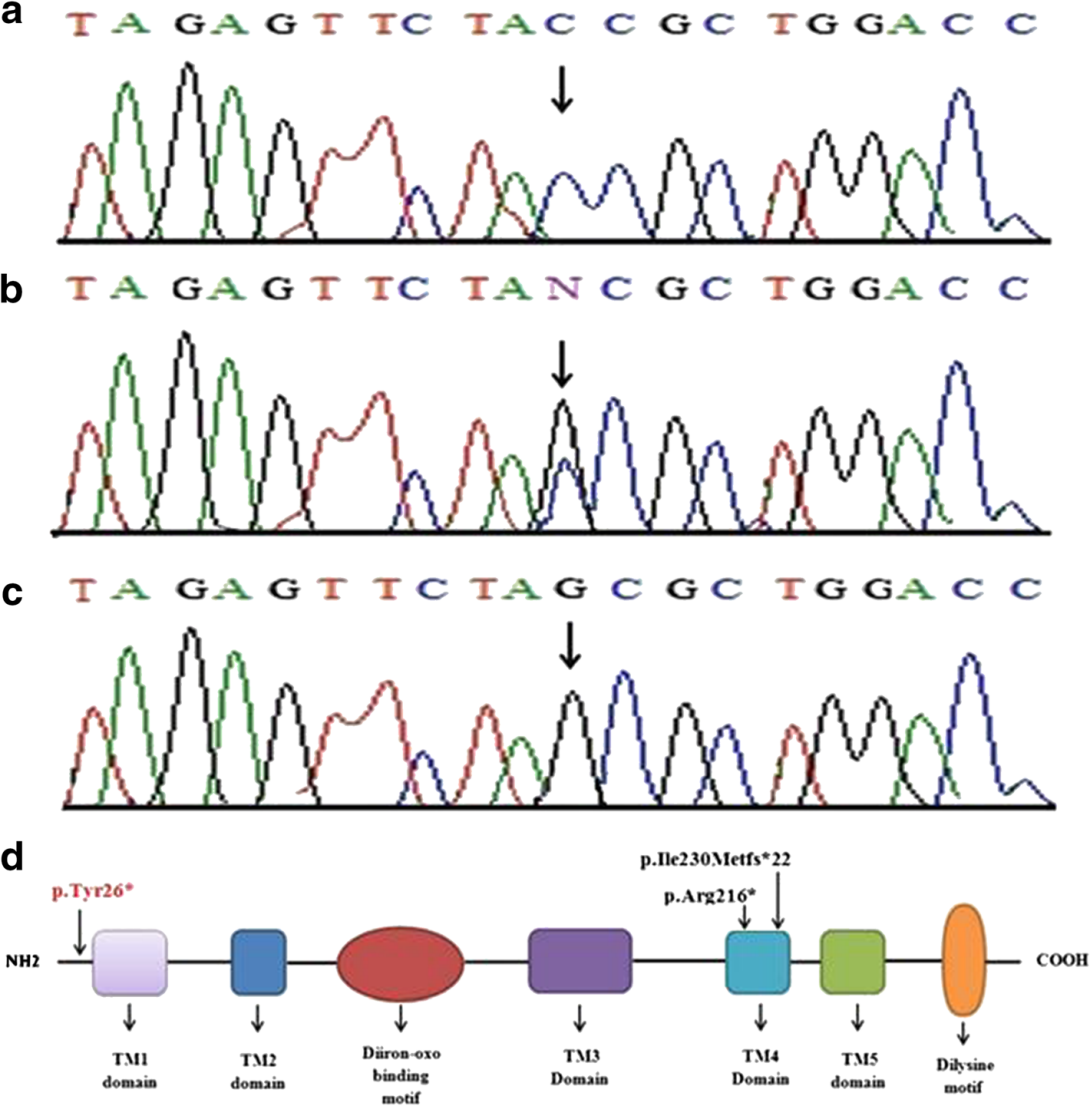
Sequence analysis of a homozygous nonsense mutation (c.78C > G; p.Tyr26*) in the gene *ELOVL4*. The upper panels **(a)** represent the nucleotide sequences in the control unaffected individual, the middle panels **(b)** in the heterozygous carrier and the lower panels **(c)** in the affected individual. Arrow indicates position of C to G transversion. **(d)** Schematic representation of the human ELOVL4 structural and functional domains. Position of the recessive mutation identified here (in red) and those reported earlier (in black) are shown. TM 1-5, Transmembrane Domain 1-5, Diiron-oxo binding domain, Dilysine motif.

Linkage in the family was established to microsatellite markers linked to the gene *ELOVL4* mapped on chromosome 6q14.1*.* Subsequently, sequence analysis of exon 1 of the gene *ELOVL4* detected a novel homozygous nonsense mutation involving C to G transition at nucleotide position 78 (c.78C > G) (Figure 3a). This resulted in substitution of a codon for tyrosine at amino acid position 26 to stop-codon (p.Tyr26*). The sequence variant, identified here, was present in the heterozygous state in the obligate carriers of the family and was not identified in 100 normal Pakistani individuals.

## Discussion

In the present investigation, we have reported clinical and molecular analysis of a consanguineous Pakistani family presenting clinical manifestations of a neuro-ichthyotic syndrome. Intra-familial phenotypic variability was observed among affected individuals of the family. Characteristic features of ichthyosis affecting different parts of the body were observed in all three affected individuals. However, other features including intellectual disability, hypertonia and spastic quadriplegia linked to the neuro-ichthyotic syndrome were observed only in one affected individual of the family. This individual died recently. Similar features were reported in two individuals with a neuro-ichthyotic syndrome by Aldahmesh et al. [8]. Clinical features including leukoplakia, prominent ears with large pinna and yellow pigmented nails, observed with variable severity, in affected individuals of the present family were not reported earlier. Other features like inguinal hernias, small testicular size, and microcephaly reported by Aldahmesh et al. [8], were not observed in affected members of our family.

Genotyping using microsatellite marker established linkage in the family to the gene *ELOVL4* on chromosome 6p14.1. Sequence analysis of the gene revealed a novel homozygous nonsense mutation (p.Tyr26*) in all three affected individuals. To date, two recessive mutations (p.Arg216*, p.Ile230Metfs*22) causing a neuro-ichthyotic syndrome [8] and three heterozygous mutations (p.Asn264Thrfs*10, p.Asn264Leufs*9, p.Tyr270*) causing Stargardt-like macular degeneration [6,11-13] have been reported in the gene *ELOVL4*.

The gene *ELOVL4*, containing six coding exons, spans 32.7 kb of genomic DNA and encode 314 amino acids protein. The ELOVL4 contains five membrane-spanning domains, a histidine cluster motif (HXXHH) involved in enzymatic activity, and endoplasmic reticulum retention signal composed of a di-lysine motif (KXKXX) at the C-terminus (Figure 3b). All the previous mutations reported in the gene *ELOVL4* were clustered in the C terminus of the protein. The novel mutation (p.Tyr26*), identified in the present study, is the only mutation located at N-terminus of the protein. This mutation is predicted to result in loss of function of the ELOVL4 protein either through nonsense mediated mRNA decay or production of a truncated protein. In the latter case, the truncated protein product lacks five transmemrane domains, a histidine cluster and a dilysine-motif. It is highly likely that the mutant ELOVL4 protein carrying the nonsense mutation (p.Tyr26*) may lack the elongase activity required for synthesis of very-long-chain fatty acids (VLCFAs) in skin and brain, and results in the abnormal localization of the protein as well.

## Conclusion

In this study we have reported only the third recessive mutation in the gene *ELOVL4* causing neuro-ichthyotic syndrome. Interestingly, analysis revealed the same mutation resulted in appearance of neurological phenotype only in one of the three affected individuals of the same family. As ELOVL4 is involved in the elongation of C26 to C28 VLCFAs, that are important components of retinal, skin and brain phospholipids and sphingolipids [1,2], any change in the structure and stability of ELOVL4 protein may alter this pathway of VLCFAs biosynthesis. One of the possible factors contributing towards phenotypic variability, observed in the three affected individuals, is alternative use of at least one of the eleven in-frame ATG codons downstream of the mutation site.

## Abbreviations

ELOVL4: Elongation of very long chain fatty acids-4; ELO: Elongase; VLCFAs: Very-long-chain fatty acids; PUFA: Polyunsaturated fatty acids; STGD3: Stargardt-like macular dystrophy type-3; IRB: Institutional Review Board; ALDH3A2: Aldehyde Dehyrogenase 3A2 gene; MIM: Mendelian inheritance in man; SLS: Sjögren-Larsson Syndrome; PCR: Polymease chain reaction; MRI: Magnetic resonance imaging; CT: Computed tomography scan.

## Competing interests

The authors declare that they have no competing interest.

## Authors’ contributions

HM carried out the molecular genetic studies and drafted the manuscript; SIR carried out DNA sequencing, collected clinical data and helped in drafting the manuscript; MT, MMM and MNK located and studied the family and collected the blood samples; SJ conducted the clinical tests and provided detailed clinical reports; WA supervised all the experiments conducted in the lab, drafted the manuscript and provided funds for the study. All authors read and approved the final manuscript.

## Pre-publication history

The pre-publication history for this paper can be accessed here:

http://www.biomedcentral.com/1471-2350/15/25/prepub
